# Growth characteristics, redox potential changes and proton motive force generation in *Thermus scotoductus* K1 during growth on various carbon sources

**DOI:** 10.3934/microbiol.2024045

**Published:** 2024-11-22

**Authors:** Hripsime Petrosyan, Karen Trchounian

**Affiliations:** 1 Department of Biochemistry, Microbiology and Biotechnology, Faculty of Biology, Yerevan State University, 0025 Yerevan, Armenia; 2 Microbial Biotechnologies and Biofuel Innovation Center, Faculty of Biology, Yerevan State University, 0025 Yerevan, Armenia; 3 Scientific-Research Institute of Biology, Yerevan State University, 0025 Yerevan, Armenia

**Keywords:** *Thermus scotoductus*, aerobic metabolism, temperature, proton motive force, pH

## Abstract

The extremophile microorganism *Thermus scotoductus* primarily exhibits aerobic metabolism, though some strains are capable of anaerobic growth, utilizing diverse electron acceptors. We focused on the *T. scotoductus* K1 strain, exploring its aerobic growth and metabolism, responses to various carbon sources, and characterization of its bioenergetic and physiological properties. The strain grew on different carbon sources, depending on their concentration and the medium's pH, demonstrating adaptability to acidic environments (pH 6.0). It was shown that 4 g L^−1^ glucose inhibited the specific growth rate by approximately 4.8-fold and 5.6-fold compared to 1 g L^−1^ glucose at pH 8.5 and pH 6.0, respectively. However, this inhibition was not observed in the presence of fructose, galactose, lactose, and starch. Extracellular and intracellular pH variations were mainly alkalifying during growth. At pH 6.0, the membrane potential (ΔΨ) was lower for all carbon sources compared to pH 8.5. The proton motive force (Δp) was lower only during growth on lactose due to the difference in the transmembrane proton gradient (ΔpH). Moreover, at pH 6.0 during growth on lactose, a positive Δp was detected, indicating the cells' ability to employ a unique energy-conserving strategy. Taken together, these findings concluded that *Thermus scotoductus* K1 exhibits different growth and bioenergetic properties depending on the carbon source, which can be useful for biotechnological applications. These findings offer valuable insights into how bacterial cells function under high-temperature conditions, which is essential for applying bioenergetics knowledge in future biotechnological advancements.

## 1. Introduction

In the realm of extremophiles, *Thermus scotoductus* stands out as a remarkable microorganism that thrives in extreme environments characterized by high temperatures, slight acidic-alkaline conditions, and mineral-rich waters [1]–[4]. It has the interest of scientists and researchers worldwide due to its exceptional adaptability and its potential applications in various biotechnological fields [5]–[9].

Thermophilic bacterial membranes undergo lipid composition changes in response to fluctuations in growth temperatures, which ensures membrane fluidity for the regulation of membrane-bound enzymes and transport systems [10],[11]. Various proposals have been reported to explain the high thermal stability of *Thermus sp*. cell membrane and proteins; therefore, a protective mechanism depends on both amino-acid composition and their secondary, tertiary structures [12],[13]. Bioenergetic properties relay on membrane composition and ion (proton or sodium ion) impermeability for energy generation, where cells keep it constant regardless of environmental conditions, although, depending on carbon sources bioenergetic indicators (redox potential, membrane potential, ΔpH) and other environmental conditions vary at different pH values [10]. It has been reported that at higher temperatures, membranes exhibit increased permeability to molecules and ions. Moreover, exergonic reactions in hydrothermal vents are considered to dominate facilitating the synthesis of organic matter [10],[14],[15].

Members of the genus *Thermus* exhibit a wide range of metabolic capabilities. While initially considered strictly aerobic, recent discoveries have shown that many *Thermus* isolates can grow anaerobically [16],[17]. *T. scotoductus* K1 strain was isolated from neutral 70 °C geothermal spring outlet [18], which is capable to use nitrate as a terminal electron acceptor and oxidize substrates anaerobically [19],[20]. While some strains utilize diverse carbohydrates, without carbon catabolite repression (co-utilization) [21], carboxylic acids, amino acids, and proteins as carbon sources, others display unique metabolic preferences, often influenced by their natural habitats. *Thermus* species' preference for low-nutrient conditions has led to their classification as facultative oligotrophs, indicating their adaptability to both low and high-nutrient environments [1]. Membrane modifications such as increase of iso-branched fatty acids also take place to improve the rigidity of the membrane, by which cell membrane respond to growth parameters, e.g. temperature, redox potential, nutrient availability [22]. Properties of *Thermus sp*. as thermophily, acidophily, chemoautothrophy are promising in fields of modern technologies for thermostable enzyme production, bioremediation and bioleaching, as well as bioenergy [23].

Genes responsible for aerobic respiration encodes numerous genes assigned to a classical electron transport chain, which shown in *T. scotoductus* SA-01 [16],[24]. The metabolism of thermophiles, compared to mesophiles, follows many of the same pathways but with some key differences. Thermophilic bacteria need to produce more energy to survive in harsh, high-temperature environments, which causes certain metabolic pathways to be adjusted: Some are activated more, while others, like the ATP-consuming Krebs cycle, may be downregulated. As a result, intermediate metabolites build up, and substrates may only be partially oxidized [11]. Membrane proteins are responsible for a wide range of substrates transportation, including ions, sugars, amino acids, and drugs [10],[25],[26]. One promising aspect of *Thermus* species metabolic diversity lies in their ability to synthesize hydrolases that break down complex organic matter [16],[27]–[29], as well as production of secondary metabolites [30].

It was shown, that in different extremophiles Δp has values in range of ~ −250 mV to −50 mV [31]. Moreover, in *T. onnurineus* NA1 ATPase dependent proton and sodium flux was determined growing on a single formate [32]. The variations in the Δp of *T*. *scotoductus* K1 under different environmental conditions are shown below, with some values falling outside the commonly reported ranges.

Within the genus *Thermus*, *T. scotoductus* is classified under the species *Thermus scotoductus* SA-01, VI-7, X-12, and others. Our aim of this article is to describe growth and bioenergetic parameter changes in aerobically cultivated *T. scotoductus* K1 strain in the presence of different carbon sources and pH values. It was shown that *T. scotoductus* can grow on various types and concentrations of carbon sources at different pHs. It is worth mentioning that high concentrations of lactose and starch did not inhibit the growth of the strain. These findings provide a significant basis for further investigation to uncover bioenergetics in thermophilic life.

## 2. Materials and methods

### 2.1. Culture media and cultivation

*Thermus scotoductus* K1 strain was kindly provided by Dr. H. Panosyan (Department of Biochemistry, Microbiology and Biotechnology, Yerevan State University, Yerevan, Armenia). It was cultivated aerobically (150 rpm in a shaking incubator—ES-20/80, Biosan, Latvia) at 65 °C up to 3:2 ratio of gas: Liquid in nutrient media containing 1 g L^−1^ peptone, 1 g L^−1^ yeast, and mineral salt solution according to ATCC medium 2293 [18]. To measure growth parameters, cells were grown in the presence of various sole carbon sources (glucose, fructose, galactose, glycerol, lactose, and starch) in a concentration-dependent manner (1–4 g L^−1^) at pH 8.5 and pH 6.0.

### 2.2. Determination of specific growth rate, pH and ORP (mV)

The specific growth rate (µ) was determined by measuring optical density (OD) at 600 nm using a UV-Vis spectrophotometer (Cary 60, Agilent, USA) [26]. An overnight bacterial culture was suspended in the prepared media to a final concentration of 1.5%. OD was measured periodically each hour. µ was calculated as the change in the mean OD value per hour during the log phase (h^−1^). External or medium pH (pH_out_), redox potential (ORP, mV), and OD were measured during growth for up to 120 hours [33]. pH_out_ was measured using a pH meter with a selective pH electrode (HI1131, Hanna Instruments, Portugal), and ORP was measured using an ORP electrode (HI3131B, Hanna Instruments, Portugal).

### 2.3. Determination of intracellular pH (pHin) and transmembrane proton gradient (ΔpH)

The use of 9-aminoacridine (9-AA) is widely employed as a fluorescent probe for determining intracellular pH (pH_in_) [34]. According to the method, 0.01 mM 9-AA is added to initiate fluorescence under 339 nm and absorption is measured at 460 nm. The light intensity was kept constant. Fluorescence decreases when cells are added to the environment. Quenching occurs with different transmembrane pH values, increasing in parallel with the extracellular pH. Bacteria grown overnight in culture media were centrifuged at 5000 xg for 10–15 minutes at 4 °C (Thermo Scientific Sorvall LYNX 6000, Germany). The pellet was resuspended in 100 mM Tris buffer containing 1 mM NaCl, 1 mM KCl, and 0.4 mM MgSO_4_. The buffer pH corresponded to the culture media pH (8.5 or 6.0). Tris buffer with different pH values was prepared in advance in 0.5-point steps (from 5.5 to 10) and assayed for each condition. First, 3 mL of buffer and 30 µL of 9-AA were mixed and measured. Then, 30 µL of the bacterial suspension was added to measure the quenching (Cary Eclipse Fluorescence Spectrometer, Agilent Technologies, USA). Probe OD values were up to 1 at the time of measuring the transmembrane pH difference [35],[36]. For measuring the external media pH (pH_out_) during bacterial growth, a pH meter with a selective pH electrode (HI1131, Hanna Instruments, Portugal) was used as described above. ΔpH was calculated as the difference between pH_in_ and pH_out_ as described by Gevorgyan et al. [35].

### 2.4. Determination of bacterial membrane potential (ΔΨ), proton motive force (Δp)

Membrane potential (ΔΨ), inside negative, was measured in bacterial suspension by TPP^+^ (tetraphenylphosphonium ion) selective electrode [37]. Overnight grown bacterialculture was centrifuged (Thermo scientific Sorvall LYNX 6000, Germany) 5000 xg for 10–15 min at 4 °C, pellet was resuspended in 100 mM Tris buffer containing NaCl 1 mM KCl 1 mM MgSO_4_ 0.4 mM, buffer pH corresponded to pH of culture media 8.5 or pH 6.0, adjusted by HCl. When TPP^+^-electrode immersed in the buffer thermostated at 65 °C, 1% volume of 10^−4^ mM TPP^+^ was added by two continuous times to fix the electrode deviation. A total of 10% volume of suspended bacteria added to buffer by following value changes until constant signals measure TPP^+^ flux into the cell depending state on membrane potential [35].

Δp was calculated as a sum of ΔΨ and ΔpH according to ΔµH+/F = ΔΨ − ZΔpH (negative value in mV) where Z is RT/F equal to 67 mV at 65 °C [38],[39].

### 2.5. Statistics

Results are presented as mean ± SD. A p-value of less than 0.05 was considered significant. Data were visualized using GraphPad Prism 8 software. Significance (p < 0.05) was determined by two-way ANOVA and Tukey's multiple comparisons test. Average data obtained from 3 independent assays are represented [40].

## 3. Results

### 3.1. Growth in presence of sole carbon sources at pH 8.5 and pH 6.0

**Figure 1.**
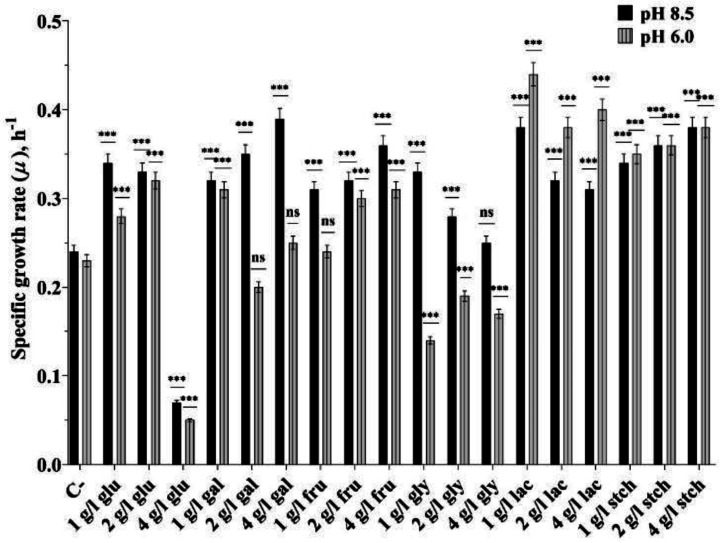
Specific growth rate (µ) of *Thermus scotoductus* K1, in presence of different concentrations of glucose (glu), galactose (gal), fructose (fru), glycerol (gly), lactose (lac), and starch (stch). For growth conditions and methods, see Materials and methods. *, **, *** signs stand for to show significance, ns—not significant, with minimum of three replicates.

**Figure 2.**
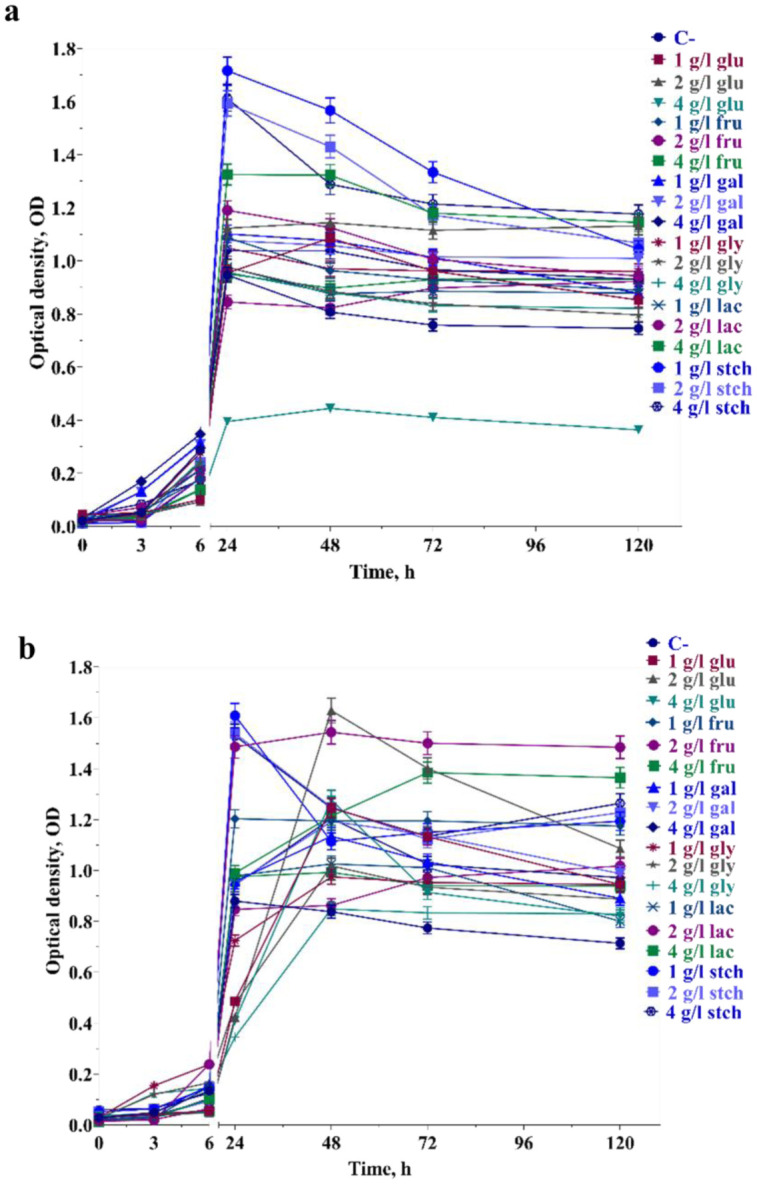
Optical density (OD) changes during the bacterial growth up to 120 h in presence of sole carbon sources at pH 8.5 (a) and pH 6.0 (b). For more information see materials and methods and legends to Figure 1.

*T. scotoductus* K1 was cultivated aerobically in the absence or presence of sole carbon sources (glucose, galactose, fructose, glycerol, lactose, and starch) at concentrations of 1, 2, and 4 g L^−1^ at pH 8.5 and pH 6.0 to observe bioenergetic and physiological differences between alkaline and acidic conditions. When bacteria were grown in culture media without any carbon source, the specific growth rate (µ) reached ~0.24 h^−1^ at pH 8.5 and ~0.23 h^−1^ at pH 6.0, which is a typical growth rate for *Thermus sp*. also shown in *T. thermophiles* HB8 [41]. However, µ varied depending on the carbon source type and concentrations (Figures 1 and 2). µ increased by ~1.4-fold in the presence of glucose at 1 and 2 g L^−1^ concentrations at pH 8.5 compared to the absence of a carbon source, while it increased by ~1.2 and ~1.4-fold at pH 6.0. Growth was inhibited in the presence of 4 g L^−1^ glucose at both pH 8.5 and pH 6.0, by ~4.8 and ~5.6-fold, respectively, compared to 1 g L^−1^ glucose. A slight inhibition of about ~1.2-fold was observed with 1 g L^−1^ glucose at pH 6.0 compared to 1 g L^−1^ glucose at pH 8.5 (Figures 1 and 2).

Similar results were obtained with 1 g L^−1^ galactose at both pHs, but not at other concentrations, where growth was inhibited by ~1.75 and ~1.56-fold at 2 g L^−1^ and 4 g L^−1^ concentrations respectively compared to pH 8.5. µ remained similar in the presence of fructose regardless of concentration and pH conditions. However, it was higher in the presence of lactose at pH 6.0 compared to pH 8.5, with no differences observed in the presence of starch. In the presence of glycerol at concentrations of 1, 2, and 4 g L^−1^, µ was inhibited by ~2.35, ~1.47, and ~1.47-fold at pH 6.0 respectively compared to pH 8.5, however, the biomass obtained reached more than OD = 1 similarly at the beginning of the stationary phase at 24^th^ hour of growth at both pHs.

Similar results were obtained with 1 g L^−1^ galactose at both pHs, but not at other concentrations, where growth was inhibited by ~1.75 and ~1.56-fold at 2 g L^−1^ and 4 g L^−1^ concentrations respectively compared to pH 8.5. µ remained similar in the presence of fructose regardless of concentration and pH conditions. However, it was higher in the presence of lactose at pH 6.0 compared to pH 8.5, with no differences observed in the presence of starch. In the presence of glycerol at concentrations of 1, 2, and 4 g L^−1^, µ was inhibited by ~2.35, ~1.47, and ~1.47-fold at pH 6.0 respectively compared to pH 8.5, however, the biomass obtained reached more than OD = 1 similarly at the beginning of the stationary phase at 24^th^ hour of growth at both pHs.

**Figure 3.**
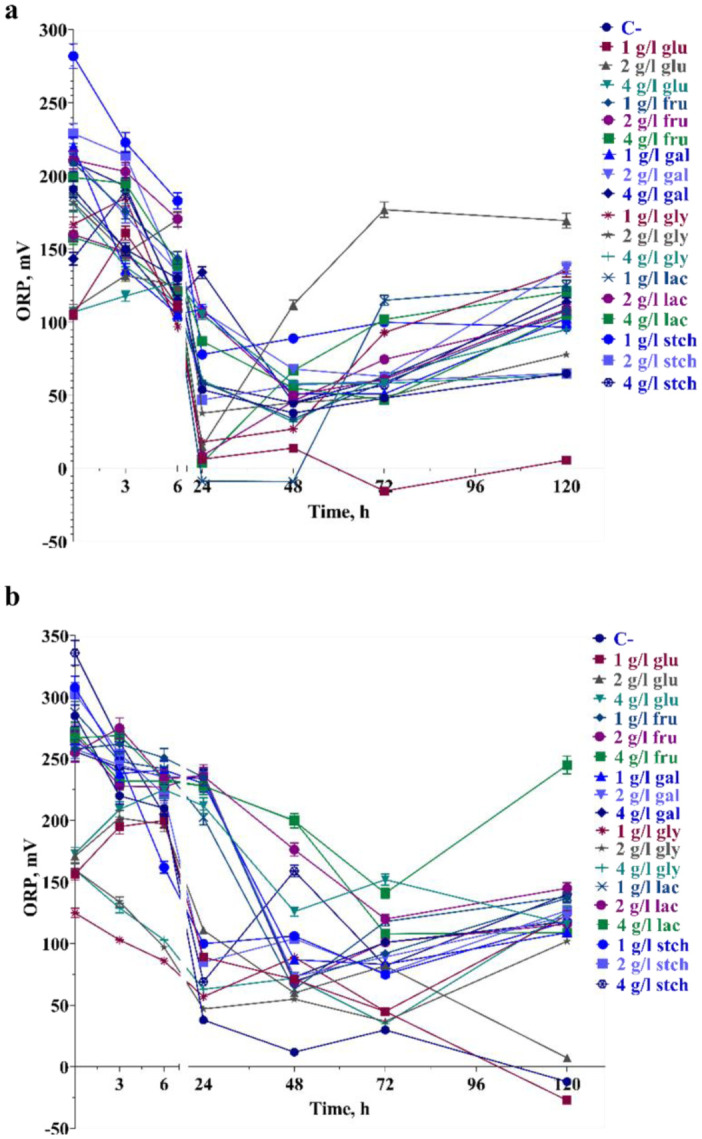
ORP (mV) value changes during the bacterial growth up to 120 h in presence of sole carbon sources at pH 8.5 (a) and pH 6.0 (b). For more information, see Materials and methods and legends to Figure 1.

It is worth mentioning that several growth measurements were assayed in the presence of sole carbon sources at a 6 g L^−1^ concentration, where growth was expected to be inhibited. However, in galactose, lactose and glycerol, biomass was produced with OD > 1 from 24–48 hours of growth (data not shown). Bacteria grown at pH 6.0 showed delayed biomass production. However, at 48 hours of growth, all above-mentioned conditions did not inhibit the final biomass production (Figure 3). During aerobic growth up to 120 hours, ORP dropped from the 6^th^ to the 48^th^ hour of growth at pH 8.5. The lowest ORP values were observed to be −15.3 and −8.9 mV in 1 g L^−1^ glucose and 1 g L^−1^ lactose utilization at the 48^th^ and 72^nd^ hour of growth respectively (Figure 3). The ORP range was 0 to +100 mV between the 24^th^ and 72^nd^ hour of growth. Interestingly, the ORP drop was slight in the presence of starch, where biomass production was not inhibited. In the presence of glucose, ORP varied across all concentrations during the growth phases. In contrast to pH 8.5, ORP drops prolonged under some conditions up to 72 hours. Meanwhile, ORP values continued to drop in 1 and 2 g L^−1^ glucose, reaching values below zero. In most cases, extracellular pH tended to become more alkaline when bacteria reached the stationary phase at pH 8.5. However, after 72 hours in glycerol and lactose-containing medium, pH remained constant or showed a 0.15-point difference in all concentrations.

**Figure 4.**
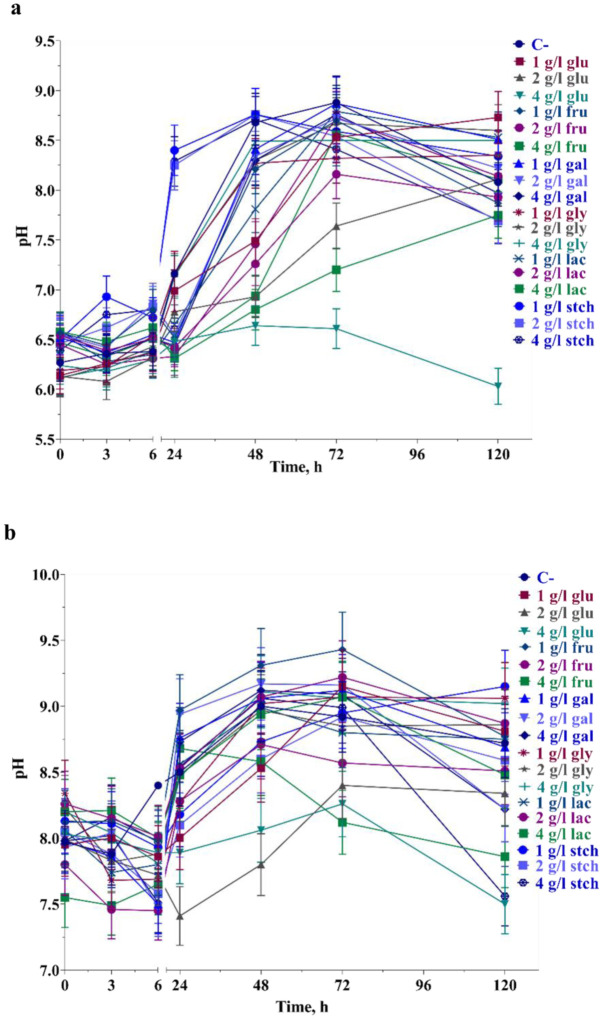
pH value changes during the bacterial growth up to 120 h in presence of sole carbon sources at pH 8.5 (a) and pH 6.0 (b). For more information, see Material and methods and legends to Figure 1.

External pH became more alkaline when bacteria were grown at pH 6.0, except with 4 g L^−1^ glucose where growth was inhibited (Figure 4). However, pH changes at pH 6.0 were greater than at pH 8.5, but the maximum point observed was less than at pH 8.5, even in the late stationary phase of growth. Acidification in early growth phases at pH 8.5 was typical in all conditions, while for pH 6.0, it was mostly observed in carbon sources with 2 and 4 g L^−1^ concentrations, with a maximum pH drop of 0.15 ± 0.02 at pH 6.0 and 0.66 ± 0.03 at pH 8.5. On the other hand, external pH became more alkaline in the presence of glycerol and starch at pH 6.0.

### 3.2. Intracellular pH, transmembrane pH gradient (ΔpH), membrane potential (ΔΨ), and proton motive force generation (Δp)

**Figure 5.**
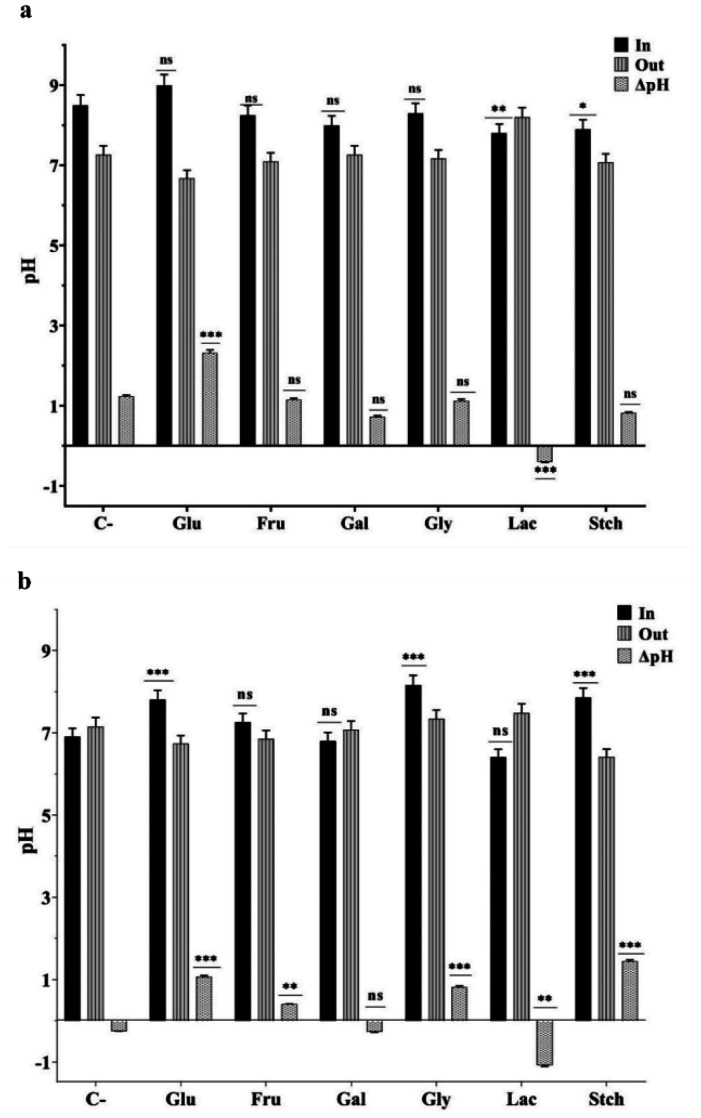
Extra- and intracellular pH values of *Thermus scotoductus* K1 grown in 2 g L^−1^ concentration of sole carbon sources at pH 8.5 (a) and pH 6.0 (b). For more information, see Materials and Methods and legends to Figure 1. *, **, *** signs stand for to show significance, ns—not significant.

**Figure 6.**
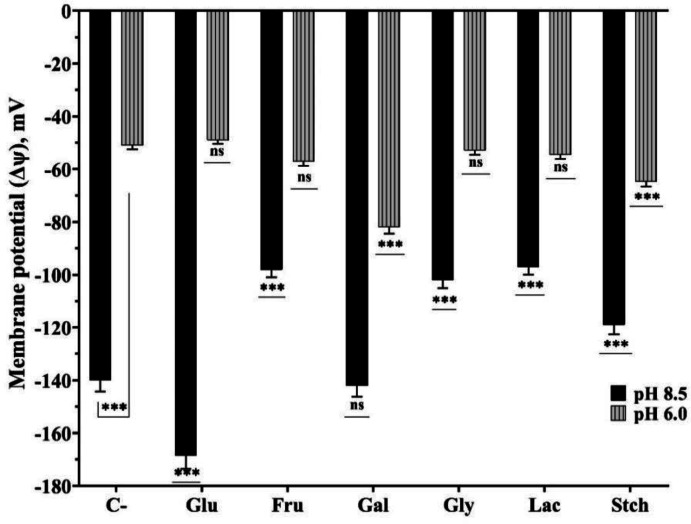
Membrane potential (ΔΨ) of *Thermus scotoductus* K1 grown in 2 g L^−1^ concentration of sole carbon sources at pH 8.5 and pH 6.0. For more information see Materials and Methods and legends to Figure 1. *, **, *** signs stand for to show significance, ns—not significant.

Intracellular pH, transmembrane pH gradient (ΔpH), and membrane potential were determined to understand the cell bioenergetics properties. Sole carbon sources were added at a concentration of 2 g/L and incubated for up to 20–24 hours. When bacteria were grown at pH 8.5, intracellular pHs were higher, with a greater ΔpH_in/out_ observed in the presence of glucose, which was approximately 2.3 (Figure 5). The lowest pH_in_ was measured in the presence of starch and galactose at pH 8.5, approximately 7.9 and 8, respectively. ΔpH_in/out_ remained similar in the presence of fructose and glycerol, as well as in the absence of a carbon source, although the absolute pH values were different. Overall, intracellular pH ranged from 8 to 9 when initially grown at pH 8.5, and from 6.8 to 8.15 when grown at pH 6.0. At 20–24 hours of growth, ΔΨ values were comparatively low at pH 6.0, ranging from −39 to −71 mV, while at pH 8.5, they varied from −95 to −170 mV depending on the presence or absence of carbon sources (Figure 6). ΔΨ was highest in the presence of glucose, approximately −170 mV at pH 8.5, and −82 mV at pH 6.0 in the presence of galactose. ΔΨ, similar to ΔpH variations, remained in similar ranges at both pH 8.5 and pH 6.0 in the presence of fructose and glycerol.

The proton motive force (Δp) was higher, especially in the presence of glucose (+324 mV), compared to the absence of a carbon source (+223 mV), and other carbon sources, except for lactose, which showed a Δp of +70 mV at pH 8.5 (Table 1). In the absence of a carbon source at pH 6.0, Δp dropped by ~6.5 fold compared to pH 8.5. However, glucose increased the Δp by ~3.5 fold at pH 6.0. Interestingly, the highest Δp (inside negative) was in the presence of starch, while lactose resulted in a Δp (inside positive).

**Table 1.** Proton motive force (Δp) calculation in given conditions of growth parameters at pH 8.5 and pH 6.0. Calculation was done at 65 °C.

Carbon source	pH 8.5	pH 6.0
No carbon source	−222.41	−34.25
Glucose	−323.94	−120.69
Fructose	−175.05	−83.8
Galactose	−190.91	−63.91
Glycerol	−177.71	−107.94
Lactose	−70.2	+17.81
Starch	−173.94	−161.18

## 4. Discussion

Reports suggest that *Thermus* species exhibit oligotrophy, where their growth can be inhibited by high substrate concentrations. This occurs because incomplete metabolism leads to the accumulation of intermediates and end products, which in turn inhibit further oxidation of precursors [1]. The presence of peptone and yeast in media provides nutrient source (organic N, some vitamins) and promotes biomass production. Therefore, the concentration used was sufficient for the strain to grow, and most common media where thermophiles are being cultured composed 0.5–1 g L^−1^ of complex organic matter, e.g., Thermus agar, R2A agar. Moreover, bacteria were able to grow in all culture media showing growth characteristics (biomass) with little variation; the stationary phase appeared between 12–48 hours of growth. Bacterial growth was also monitored after the log phase, up to 120 hours. A concentration of 4 g L^−1^ glucose inhibited bacterial growth at pH 8.5, which proves oligotrophy of *Thermus sp*. toward sugar concentration. Despite this, bacterial biomass was produced in most cases, with surprising OD values reaching up to ~1.7 in 1 g L^−1^ starch, even though reports suggest that less biomass is typical for *Thermus sp*. and growth is inhibited in most sugars, which is shown for only glucose among other tested substrates [16]. Furthermore, biomass in a stationary phase was achieved to similar ODs in higher concentrations of peptone and yeast by Babák et al. [4]. As shown, µ was affected at pH 6.0, but not biomass production, indicating that the bacteria exhibited tolerance to acidic environments, which is typical for hot spring inhabitants [42].

Changes in extracellular physicochemical parameters are crucial for understanding bacterial metabolism and its type and direction, which can inform the study of thermophilic physiology under different growth conditions [43]. ORP was more reduced in the presence of glucose and lactose compared to samples without any carbon source. In the presence of glucose, ORP varied across all concentrations during the growth phases, indicating different rates of metabolic processes.

Measuring external pH is crucial for understanding the types of predominant compounds present in the media, as it provides insights into how pH variations influence the microbial environment's composition and dynamics. However, pH changes at pH 6.0 were more pronounced than at pH 8.5, but the maximum pH observed was lower at pH 6.0. Alkalinization was expected in all conditions, as the bacteria were cultivated aerobically, and acids and acidifying compounds were assumed to be in small amounts. Acidification in the early phases of growth at pH 8.5 was typical in all conditions, with similar trends for pH 6.0, especially at carbon sources with 2 and 4 g L^−1^ concentrations. The maximum pH drop was 0.15 ± 0.02 at pH 6.0 and 0.66 ± 0.03 at pH 8.5. Conversely, external pH was alkalified in the presence of glycerol and starch at pH 6.0. Regulation of pH and the transmembrane pH gradient (ΔpH) is crucial for enzyme synthesis and metabolism, which can reveal unique properties at high temperatures.

Intracellular pH, transmembrane pH gradient (ΔpH), and membrane potential are key determinants of cellular electrochemical homeostasis. These parameters intricately orchestrate cellular functions by influencing enzymatic activities, ion transport, and sensitivity to compounds, cell signaling, and molecular dynamics. Despite survival in media with a wide range of pH, bacteria have elaborate mechanisms to maintain a constant neutral intracellular pH under various growth conditions [43]–[45]. This significant difference implicates active physiological processes such as enzymatic activity, generation of proton motive force, and organic acid production [43],[44].

Overall, bacteria kept intracellular pH with slight variations, which is considered constant. Similar data has been observed in both thermophilic anaerobic and mesophilic bacteria [44]. The intracellular pH ranged from 8 to 9 when initially grown at pH 8.5, and from 6.8 to 8.15 when grown at pH 6.0. Hence, pH stress response mechanisms function differently, with the main role being the initial pH value of the environment. It has been shown that *Thermus* representatives can express β-galactosidase for lactose hydrolysis [46]–[48], leading to unique pH variations in both pH 8.5 and pH 6.0, where intracellular pH was lower than extracellular (Figure 5). Low ΔpH values can be explained by low permeability to organic matter and low ion flux activity.

To fully understand the bioenergetics of *Thermus scotoductus* K1, more data and investigations are needed for other species, such as how bacterial ΔpH_in/out_ varies during the log or late stationary phases of growth. High ΔΨ or ΔpH drives the proton motive (hydrogen or sodium ion) force for energy generation, affecting ATP synthesis and other energy-dependent processes. Whether the cytoplasmic pH is higher or lower than the external pH, the ΔpH contributes to or detracts from the total proton motive force. As shown, lower ΔΨ indicates higher permeability for ion and compound fluxes through the membrane, suggesting that Δp generation (energy generation) could be based on either membrane potential or outer and inner pH differences. For example, Δp variation at pH 8.5 and pH 6.0 depends on ΔΨ but not ΔpHin/out in the presence of galactose. The presence of lactose showed Δp inside positive at pH 6.0, in contrast to pH 8.5, indicating that endergonic reactions and metabolism are dominant in the cells, and ion and solute permeability is lower. This suggests that bacteria have a unique survival strategy and can regulate and maintain cell energy balance.

Consequently, significant variations in intracellular pH and membrane potential of *Thermus scotoductus* K1 under different culture conditions are experimentally observed. Higher intracellular pH at pH 8.5, especially with glucose, indicates distinct physiological processes influenced by environmental factors. The findings offer valuable insights into the adaptive responses of *Thermus scotoductus* K1 and changes in bioenergetics parameters depending on diverse growth conditions. Calculations of Δp at pH 8.5 in most carbon sources were higher than in mesophilic bacteria [36], except in the presence of lactose. However, the results indicate lower metabolic processes at pH 6.0, where the presence of lactose resulted in Δp being inside positive, suggesting a unique survival strategy for bacteria and their ability to regulate and maintain cell energy balance. This physiological phenomenon is important for further analysis to understand the role of carbon sources in acid formation and to compare anaerobic/aerobic metabolism to detect if similar strategies are manifested.

## 5. Conclusion

To conclude, this study investigated the growth and bioenergetic responses of *Thermus scotoductus* K1 to different carbon sources and pH conditions. Extracellular bioenergetic parameters, such as ORP and pH changes, reflect the metabolic state and physiological responses of *T. scotoductus* K1, highlighting the intricate relationship between environmental conditions and bacterial metabolism in extreme environments. The investigation of intracellular pH (pH_in_) and membrane potential (ΔΨ) under varying conditions provides insight into the bacterium's physiological responses. The significant elevation of intracellular pH in the presence of glucose at pH 8.5 suggests active physiological processes, including enhanced enzymatic activity and proton motive force generation. Notably, pH stress response mechanisms operate differently depending on the initial pH of the environment. Further studies could reveal specific mechanisms and implications of these findings for the bacterium's survival strategies and their potential applications in biotechnological fields.

## Use of AI tools declaration

The authors declare they have not used Artificial Intelligence (AI) tools in the creation of this article.
